# *Streptococcus suis* sortase A is Ca^2+^ independent and is inhibited by acteoside, isoquercitrin and baicalin

**DOI:** 10.1371/journal.pone.0173767

**Published:** 2017-03-20

**Authors:** Fuguang Chen, Fang Xie, Baoling Yang, Chengcheng Wang, Siguo Liu, Yueling Zhang

**Affiliations:** 1 Division of Bacterial Diseases, State Key Laboratory of Veterinary Biotechnology, Harbin Veterinary Research Institute, Chinese Academy of Agricultural Sciences, Xiangfang District, Harbin, PR China; 2 College of Animal Science and Veterinary Medicine, Shenyang Agricultural University, Shenyang, PR China; 3 College of Veterinary Medicine, Northeast Agricultural University, Xiangfang District, Harbin, PR China; Universidad de Santiago de Compostela, SPAIN

## Abstract

Sortase A (SrtA) has long been recognized as an ideal drug target for therapeutic agents against Gram-positive pathogens. However, the SrtA of *Streptococcus suis* (Ss-SrtA), an important zoonotic agent, has not been studied. In this study, the enzymatic properties of Ss-SrtA were investigated, and inhibition of Ss-SrtA by natural products was evaluated. Ss-SrtA was expressed and purified. The purified recombinant Ss-SrtA had maximal activity at pH 6.0–7.5, 45°C, and showed a *K*m of 6.7 μM for the hydrolysis of substrate abz-LPATG-dnp. Different from *Staphylococcus aureus* SrtA (Sa-SrtA) which is stimulated by Ca^2+^, Ss-SrtA was observed to be Ca^2+^ independent. Structural analysis showed that salt bridges formed between K111 and D180 in Ss-SrtA replaced the function of Ca^2+^ in Sa-SrtA to stabilize the substrate-binding cleft. Site-directed mutagenesis identified H126, C192 and R200 as the key residues of Ss-SrtA active site. To discover potential inhibitors, the percent inhibition of sortase activity by natural products was measured. Among these selected natural products, acteoside, isoquercitrin and baicalin were discovered as novel SrtA inhibitors, with IC_50_ values of 36.3 ± 1.3 μM, 100.0 ± 1.3 μM and 85.4 ± 1.5 μM, respectively. The inhibitory effects of these three natural products were further confirmed on endogenous Sa-SrtA. Using a previously established *S*. *aureus* model with a fluorescent-labeled Sa-SrtA substrate, acteoside, isoquercitrin, and baicalin showed 86%, 28% and 45% inhibition on endogenous Sa-SrtA activity, respectively. Overall, these findings shed new light on enzymatic properties, Ca^2+^-independent catalytic mechanism and potential inhibitors of Ss-SrtA.

## Introduction

*Streptococcus suis* is one of the most important bacterial pathogens in pigs, causing major economic losses to the swine industry worldwide [1]. It is also an emerging zoonotic agent of human meningitis and streptococcal toxic shock-like syndrome [1]. *S*. *suis* is receiving growing attention not only for its role in increasingly reported severe infections in humans but also for its increasing resistance to antibiotics. High rates of *S*. *suis* resistance to tetracyclines, macrolides, β-lactams, aminoglycosides, trimethoprim-sulfamethoxazole, chloramphenicol, and fluoroquinolones have been frequently reported in pig isolates worldwide [2, 3]. Vaccines are being developed to prevent *S*. *suis* infection, such as whole-cell bacterins, autogenous bacterins in piglets, and live-attenuated vaccines. However, their protection effects are thus far unsatisfactory [4], stressing the urgent need for the study of novel therapeutic strategies against *S*. *suis* infection.

Gram-positive pathogenic bacteria display surface proteins that play critical roles in adhesion and invasion of host cells or evasion of host-immune responses [5]. Many of these proteins are covalently linked to the cell wall peptidoglycan through C-terminal sorting signal with the conserved LPXTG motif [6]. Sortase A (SrtA) is a membrane-associated transpeptidase responsible for the anchoring of these surface proteins to the cell wall by recognition of the LPXTG motif [6]. Therefore, SrtA plays a critical role in Gram-positive bacterial pathogenesis and is considered a promising anti-infective target. The other important feature of SrtA is that it is not required for bacterial growth, thus its inhibitors will not exert selective pressures to promote the development of antibiotic resistance [7]. Furthermore, SrtA resides on the extracellular side of the cell membrane and it increases the chance to interact with inhibitors [8]. Therefore, SrtA is an attractive drug target for the development of anti-infective drugs.

So far, detailed structural studies have been limited to SrtAs from *S*. *aureus* (Sa-SrtA) [9–11] and *S*. *pyogenes* (Sp-SrtA) [12]. These sortases share a similar 8 stranded β-barrel-fold structure despite of sequence diversity. The Cys, His and Arg residues clustered at the center of a long cleft are identified as the key catalytic residues for these sortases [13]. The main enzymatic difference between Sa-SrtA and Sp-SrtA is their dependence on Ca^2+^. It has been found that Ca^2+^ stimulates the activity of Sa-SrtA by 8-fold, whereas the activity of Sp-SrtA is not promoted by Ca^2+^ [12]. This has been explained by the different residue arrangements of the β3/β4 loop and β6/β7 loop in SrtA structures [11, 12]. Over the past decade, useful investigations have been performed to identify inhibitors of Sa-SrtA to combat the alarming increase in antimicrobial resistance, and promising inhibitor compounds have been discovered [13]. The results show that natural products are good resource for SrtA inhibitors.

In the case of *S*. *suis*, a mutant lacking *srtA* fails to display surface proteins and is defective in the establishment of infections [14, 15]. Genome sequencing reveals that *S*. *suis* encodes 33 surface proteins with the LPXTG sorting signal, which fulfill diverse functions during infection. Therefore, SrtA plays a critical role in *S*. *suis* pathogenesis, and SrtA inhibitors may consequently be promising candidates for the treatment and/or prevention of *S*. *suis* infections. In this study, we biochemically characterized Ss-SrtA. By screening 11 natural products, new promising Ss-SrtA inhibitors were discovered.

## Materials and methods

### Bacterial strains, plasmids and growth conditions

The bacterial strains and plasmids used in this study are listed in Table 1. *S*. *suis* strains were cultured in Todd–Hewitt broth (THB; Oxoid Ltd.) without shaking or Todd–Hewitt broth agar plates at 37°C with 5% CO_2_. *E*. *coli* and *S*. *aureus* strains were grown in Luria-Bertani (LB) medium at 37°C with shaking at 200 rpm or plated on LB agar. If required, cultures were supplemented with antibiotics at the following concentrations: erythromycin, 3 μg ml^−1^ for *S*. *aureus*; ampicillin, 100 μg ml^−1^ for *E*. *coli*.

**Table 1:** Bacterial strains and plasmids used in this study.

Bacterial strains	Description	Source or reference
*S*. *suis*		
05ZYH33	A highly virulent strain isolated from a dead patient with streptococcal toxic shock syndrome	[16]
*S*. *aureus*		
Newman D2C	Wild-type SrtA positive; non-hemolytic; coagulase negative	ATCC
Λ*SrtA*	The deletion mutant of *srtA* with background of *S*. *aureus* Newman D2C	[17]
*E*. *coli*		
DH5α	Cloning host for maintaining the recombinant plasmids	Tiangen
BL21(DE3)	Expression host for exogenous protein production	Tiangen
Plasmids		
pET22b(+)	His-tag fusion expression vector; Amp^R^	Novagen
p22SsSrtA	A recombinant vector with the background of pET-22b(+), designed for expression of SrtA; Amp^R^	This study

### Bioinformatics analysis

The signal peptide was predicted by SignalP 3.0 [18]. Multiple protein sequences were aligned using ClustalW [19], and further processed with the ESPript programs [20]. Protein structure homology modeling was performed using the SWISS-MODEL Workspace (http://swissmodel.expasy.org/). Structural alignment, analysis and graph preparation were performed with PyMOL [21].

### Protein expression and purification

The DNA sequence encoding Ss-SrtA (GenBank No. YP_001198440) residues N24-Q235 was cloned into the *Nde* I-*Xho* I restriction sites of the pET22b vector, yielding p22SsSrtA. The *E*. *coli* BL21 (DE3) strain was transformed with the recombinant plasmid and grown in LB medium containing 100 μg mL^-1^ ampicillin at 37°C with continuous shaking. When the OD_600nm_ reached 0.4–0.6, Ss-SrtA expression was induced by isopropyl β-D-thiogalactopyranoside (IPTG) with a final concentration of 0.5 mM. Cells were grown for 5 h before harvesting by centrifugation. For protein purification, cell pellets were suspended in lysis buffer (50 mM Tris·HCl, 150 mM NaCl, 5 mM imidazole, pH 7.5), lysed by sonication, and then centrifuged at 12,000 rpm for 30 min at 4°C. The supernatant was then loaded onto a HisTrap HP 5-mL column (GE Healthcare), subsequently rinsed with lysis buffer and wash buffer (50 mM Tris·HCl, 150 mM NaCl, 40 mM imidazole, pH 7.5), and then rinsed with elution buffer (50 mM Tris·HCl, 150 mM NaCl, 150 mM imidazole, pH 7.5). Subsequent purification was performed on FPLC by gel filtration (50 mM Tris·HCl, 150 mM NaCl, pH 7.5) on HiLoad 16/60 Superdex 75 column (GE Healthcare). SDS-PAGE was performed with 12% (w/v) polyacrylamide gels. The protein concentration was measured by the absorbance at 280 nm using a calculated extinction coefficient: 30,370 M^-1^cm^-1^.

### Site-directed mutagenesis

Plasmid p22SsSrtA was used as the template for the introduction of desired mutations. The mutation was introduced by PCR using the appropriate primers listed in Table 2. After PCR, the amplified plasmids were digested overnight at 37°C with *Dpn* I and then transformed into *E*. *coli* DH5α. Mutants H126A, C192A, and R200A were confirmed by DNA sequencing. The mutants were expressed and purified as described above for Ss-SrtA.

**Table 2:** Oligonucleotide primers used in this study.

Primers	Primers sequence(5’-3’) ^α^	Functions
srtA_F	CGCCATATGAATTTTATTATCGGCTGGAA	For expression of Ss-SrtA in *E*. *coli*
srtA_R	CCGCTCGAGTTGTCCATAATCATACTGAT	
H126AF	GAAATTATGCGTTGGCCAGCGCCCATATTTTTG	For expression of Ss-SrtA H126A in *E*. *coli*
H126AR	GCGCTGGCCAACGCATAATTTCCTTTCCCCATCTC	
C192AF	GCTACGGACTATTATGCTACGCAACGTATTGTTG	For expression of Ss-SrtA C192A in *E*. *coli*
C192AR	GCGTAGCATAATAGTCCGTAGCTGTCACTAACGTAAC	
R200AF	CGGACTATTATGCTACGCAAGCTATTGTTGTA	For expression of Ss-SrtA R200A in *E*. *coli*
R200AR	GCTTGCGTAGCATAATAGTCCGTACATGTCACTA	

^α^ Underlined nucleotides denote enzyme restriction sites.

### Activity of Ss-SrtA

Ss-SrtA activity was monitored in 96-well black microtiter plates (PerkinElmer) using an internally quenched fluorescent peptide substrate abz-LPATG-dnp (Shanghai GL Biochem) [21]. For standard conditions, each well contained 2.5 μM purified recombinant Ss-SrtA in 200 μL of 50 mM Tris·HCl and 150 mM NaCl, pH 7.5. The reaction was initiated by the addition of 4 μL of 1 mM abz-LPATG-dnp. The increase in the fluorescence intensity was continuously monitored at 37°C for 30 min using an EnSpire Multilabel Reader (PerkinElmer) at an excitation wavelength of 309 nm and emission wavelength of 420 nm. The fluorescence was plotted against time and the initial velocity (V_0_) was calculated as the slope of the linear portion of the curve.

For *K*_*m*_ determination, final substrate concentrations ranging from 0.375 μM to 64 μM were used. *K*_m_ was determined by nonlinear regression using the GraphPad Prism software.

### Effects of temperature, pH and ions on Ss-SrtA activity

The optimal pH was determined under the standard conditions at the indicated pH. The following buffers were used: buffer A (50 mM acetic acid, 50 mM MES, 20 mM Tris, 150 mM NaCl) for pH 3.55–7.5, and buffer B (50 mM MES, 20 mM Tris, 50mM CHES, 150 mM NaCl) for pH 5.5–11.0. The pH stability was determined by incubating Ss-SrtA at the indicated pH at 37°C for 60 min. The residual activities were measured under the standard condition. The activity of the Ss-SrtA maintained at 4°C, and pH 7.5 was set as 100% activity. The optimal temperature was determined under the standard condition at the indicated temperatures. The temperature stability was determined by incubating Ss-SrtA at the indicated temperatures and, pH 7.5 for 60 min. The residual activities were measured under the standard condition. The effects of ions were determined by incubating Ss-SrtA with 5 mM metal ions in 50 mM Tris·HCl and 150 mM NaCl, pH 7.5, at 37°C for 60 min. The residual activities were measured under the standard condition. The Ss-SrtA activity under standard condition was set as 100% activity.

### Inhibition of Ss-SrtA activity by natural products

All natural products were dissolved in DMSO to a concentration of 40 mM as a stock solution. For the 200 μL reaction, Ss-SrtA was mixed with each natural product in 50 mM Tris·HCl and 150 mM NaCl, pH 7.5, to give a final concentration of 200 μM. The reaction mixture was pre-incubated for 10 min at 25°C, and catalysis was initiated by adding 4 μL of 1 mM abz-LPATG-dnp. The V_0_ obtained in the absence of inhibitor was taken as 100% and designated as the control value, and the initial inhibitory velocity (V_i_) was obtained in the presence of natural products. The percent inhibition was calculated according to the following equation: inhibitory rate = (1 − V_i_/V_0_) × 100%. Compounds with inhibitory rates against Ss-SrtA greater than 50% were used for IC_50_ determination. For IC_50_ calculations, each natural product at 7 concentrations (ranging from 3 to 200 μM) was pre-incubated with Ss-SrtA, and inhibitory rates were determined as described above. IC_50_ values were determined using a sigmoidal dose–response with the GraphPad Prism software. Triplicate measurements were taken for each data point.

### Determination of Minimum Inhibitory Concentration (MIC) of natural products

Wild-type *S*. *suis* were cultured in THB medium in a 96-wells plate in the presence of serial dilutions (0.24 to 500 μM) of natural products at 37°C with 5% CO_2_. After 24 hours, the wells were visually inspected, and the first clear well was designated as the MIC. All experiments were repeated independently three times.

### Detection of the inhibitory effects of acteoside, baicalin, and isoquercitrin using an established peptide-incorporation model

To date, there has been a need for a good model to detect the activity of endogenous Ss-SrtA in live *S*. *suis* cells. Very recently, such a model was developed in *S*. *aureus* for the incorporation of fluorescent-labeled peptide to the cell wall by SrtA [22]. As the amount of peptide incorporated was found to be dependent upon the activity of SrtA and could be conveniently analyzed by flow cytometry, it may also be a useful model to evaluate the effects of SrtA inhibitors against endogenous SrtA. Here, we utilized this model to determine the inhibitory effects of acteoside, baicalin, and isoquercitrin on SrtA activity with live cells.

Incorporation of the fluorescent peptide (FITC-conjugated AKKSELPETGGEESTNKRRNKKNHK, designated substrate 5; Shanghai GL Biochem) by endogenous SrtA was performed as described with slight modifications [22]. In brief, overnight cultures of *S*. *aureus* were diluted 1:100 in fresh LB medium and grown at 37°C with continuous shaking (200 rpm) to the late stationary growth phase. After collection by centrifugation, bacteria were washed three times with PBS and then resuspended in SrtA buffer (50 mM Tris, 150 mM NaCl, 10 mM CaCl_2_, pH 7.5). Rather than immediately initiation of the peptide incorporation by adding substrate 5, as described previously [22], here, the cell suspension was aliquoted and incubated with or without 200 μM inhibitors for 1 h. Then, using a final volume of 100 μL in 96-well plates, reactions were initiated by adding 125 μM substrates 5, and then incubated in the dark for 17 h at 37°C with shaking. Bacteria were collected by centrifugation and washed with PBS. After treatment with 1% SDS at 60°C for 5 min, bacteria were collected by centrifugation and washed with PBS. The washed bacteria were resuspended in 200 μL of 4% formaldehyde in PBS and then incubated for 15 min at room temperature. Bacteria were centrifuged and washed with PBS and then resuspended in 500 μL of PBS; bacteria-associated fluorescence was measured on a FACS FC 500 flow cytometer and analyzed using CXP Software (Beckman Coulter, Brea, CA, USA).

## Results

### Expression and purification of Ss-SrtA

**Fig 1:**
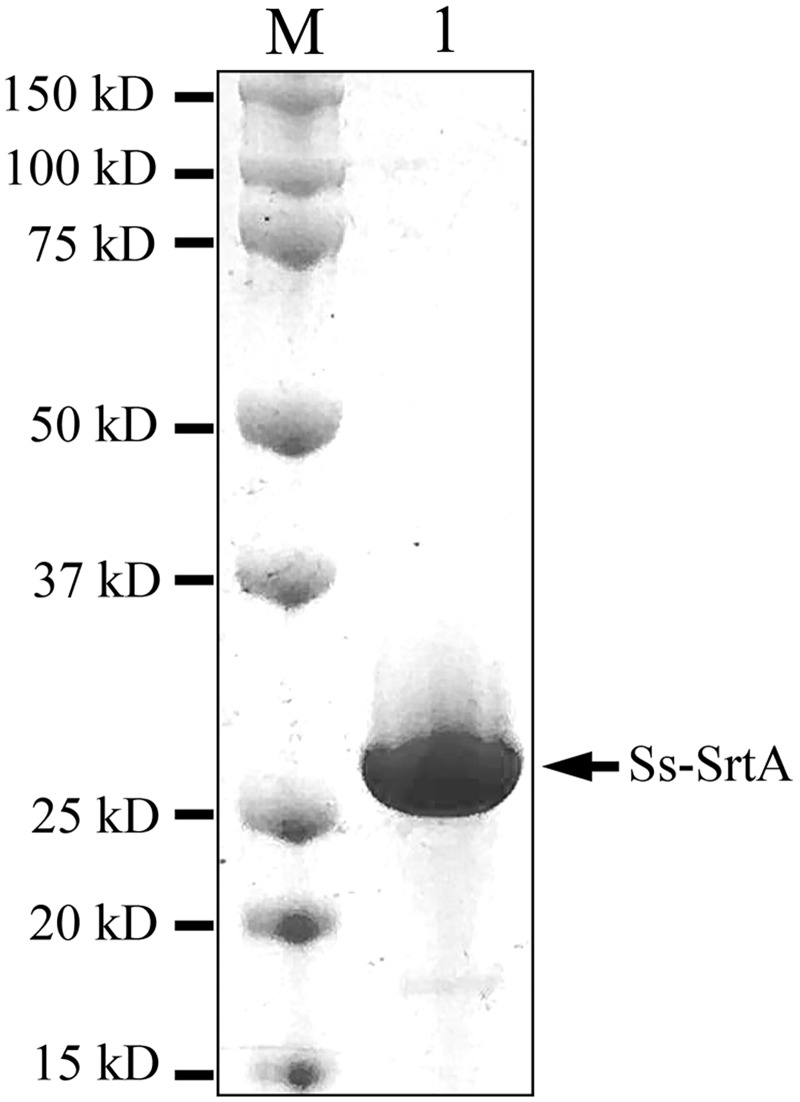
SDS-polyacrylamide gel showing the purified Ss-SrtA expressed by *E*. *coli*.

Ss-SrtA was expressed in *E*. *coli* and purified (Fig 1). SDS-PAGE showed that purified recombinant Ss-SrtA had a molecular weight of approximately 25 kDa. This was consistent with the calculated molecular weight of the recombinant Ss-SrtA, which contained Ss-SrtA residues N24-Q235 and a 6 residue C-terminal His-tag.

### Effects of temperature and pH on Ss-SrtA activity and *K*m to abz-LPATG-dnp

**Fig 2:**
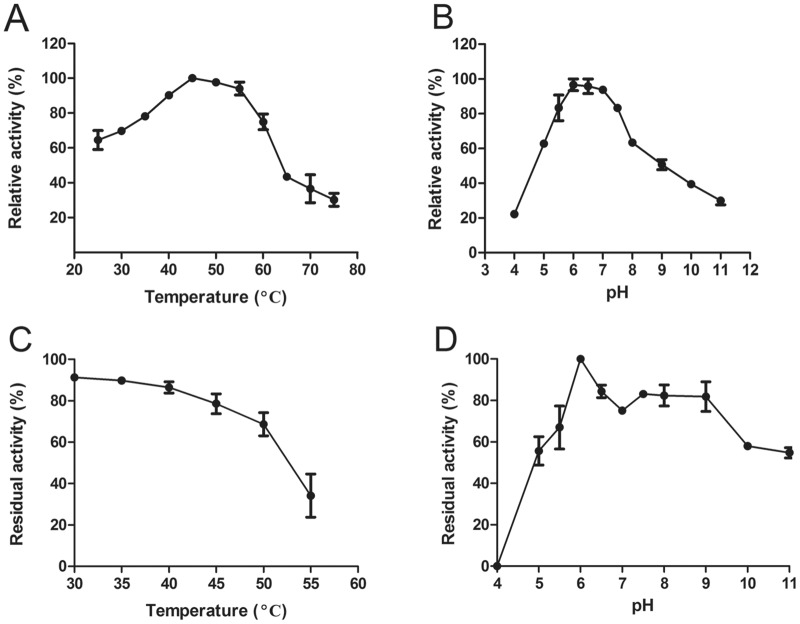
Effects of temperature and pH on the enzyme activity and stability. (A) (B). Optimum temperature and pH. (A). The optimum temperature was assayed under standard conditions at the indicated temperatures. Under standard conditions, the activity was assayed in 50 mM Tris·HCl, 150 mM NaCl, pH 7.5, using 20 μM abz-LPATG-dnp as the substrate, and the increase in fluorescence was continuously monitored at 37°C for 30 min. (B). The optimum pH was assayed under the standard condition at the indicated pH. (C) (D) Temperature and pH stability. (C). Temperature stability was determined by incubating the enzyme at the indicated temperatures, at pH 7.5 for 60 min. The residual activities were measured under the standard condition. (D). The pH stability was determined by incubating the enzyme at the indicated pH values at 37°C for 60 min. The residual activities were measured under the standard condition. The *error* bars represent the standard deviation of three independent experiments.

**Fig 3:**
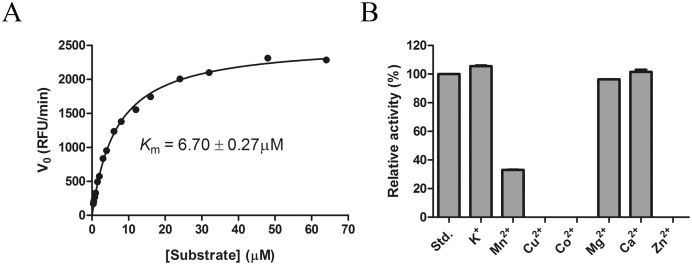
*K*_m_ calculation and effects of metal ions. (A). *K*_m_ calculation for Ss-SrtA with abz-LPATG-dnp used as a substrate. For *K*_*m*_ determination, final substrate concentrations ranging from 0.375 μM to 64 μM were used. The *K*_m_ was determined from the Michaelis–Menten equation using the GraphPad Prism software. (B). Effects of metal ions on the activity of Ss-SrtA. The assays were carried out by incubating Ss-SrtA with 5 mM metal ions in 50 mM Tris·HCl, 150 mM NaCl, pH 7.5 at 37°C for 60 min. The residual activities were measured under standard conditions.

The effects of temperature and pH on Ss-SrtA were examined. As shown in Fig 2, the optimum temperature for the enzymatic activity was 45°C (Fig 2A) and the optimum pH was 6–7.5 (Fig 2B). The enzyme retained at least 70% activity after 1 h of incubation at various temperatures up to 45°C (Fig 2C). The enzyme was stable (70% residual activity) over 60 min at pH values from 5.5 to 9 (Fig 2D). The *K*_m_ of Ss-SrtA for the hydrolysis of an internally quenched fluorescent peptide substrate abz-LPATG-dnp was 6.70 μM (Fig 3A).

### Ss-SrtA was Ca^2+^ independent

To investigate whether metal ions affected Ss-SrtA activity, the activities of Ss-SrtA in the presence of different metal ions were determined. As shown in Fig 3B, the cations Mg^2+^, Ca^2+^, K^+^ showed no significant effect on the activity of Ss-SrtA. In contrast, Ss-SrtA was completely inactivated by Cu^2+^, Co^2+^, and Zn^2+^. As well-known heavy metal ions, Cu^2+^, Co^2+^ and Zn^2+^ might inhibit the activity of Ss-SrtA by reacting with the functional group of Ss-SrtA and destructing the enzymatic structure. Partial inactivation by Mn^2+^ was observed. It is previously reported that the activity of Sa-SrtA is stimulated by Ca^2+^ [23]. However, the activity of Ss-SrtA was Ca^2+^ independent. The presence of Ca^2+^ did not affect catalytic efficiency of Ss-SrtA. The similar Ca^2+^-independency was also observed for Sp-SrtA [12].

### Model of the structure of Ss-SrtA

**Fig 4:**
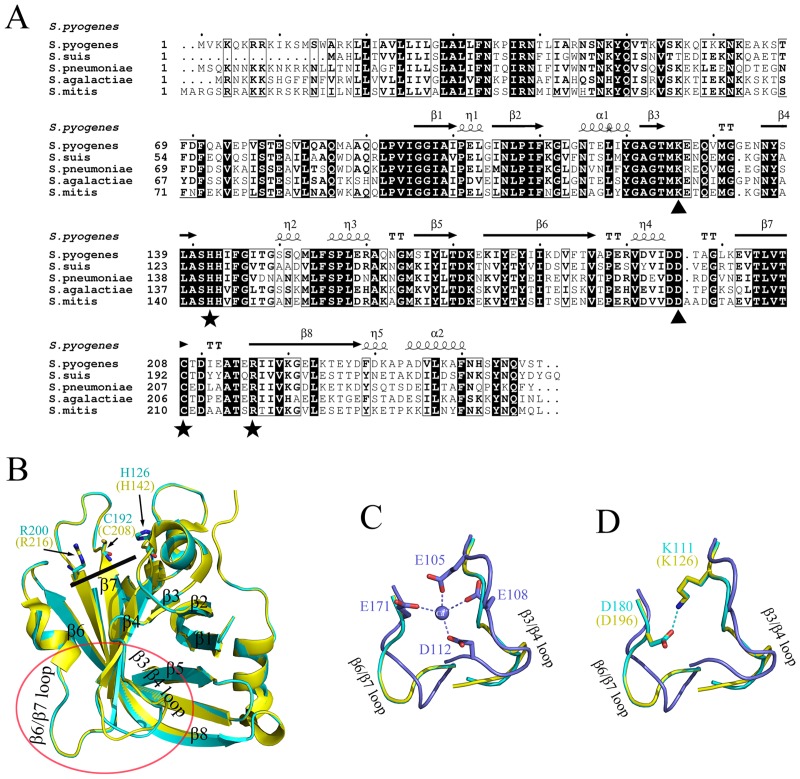
Structure alignments of Ss-SrtA and its similarities. (A). Primary structure alignment. The alignment was performed with Clustal X, and the figure was produced using ESPript. Strictly conserved residues are highlighted in black, and conservatively substituted residues are boxed. The secondary elements of Sp-SrtA (helices (α), strands (β) and 3_10_ helices (η), turn(T)) are shown above the alignment. The residues equivalent to the active site residues are indicated by solid stars, and the residues predicted to play roles in Ca^2+^-independence are indicated by solid triangles. (B). 3D-structure superposition of Sp-SrtA (yellow) structure and simulated Ss-SrtA (cyan) structure. The α-helices and β-strands that belong to the 8-stranded β-barrel-fold motif are numbered. Five key residues are shown as stick models. The substrate-binding cleft is indicated by a bold black line. β3/β4 and β6/7 loops corresponding to the loops surrounding Ca^2+^ in Sa-SrtA are cycled by red line. (C) (D). Comparison of the β3/β4 and β6/7 loops of Ss-SrtA (cyan), Sp-SrtA [12] (yellow) and Sa-SrtA [10] (purple). (C). Residues involved in Ca^2+^-binding in Sa-SrtA are shown as stick models. (D). Residues involved in Ca^2+^ independence in Ss-SrtA and Sp-SrtA are represented as stick models.

Ss-SrtA consisted of 235 amino acids and exhibited high sequence similarity to that of both *S*. *pyogenes* and *S*. *pneumoniae* (60% identity). As shown in Fig 4A, sequence alignments of these sortases were performed. The alignments revealed that H126, C192 and R200 were aligned well with active sites residues H142, C192 and R216 of Sp-SrtA, respectively, suggesting that these three residues might be the key catalytic residues in Ss-SrtA. To further explore the structural features of Ss-SrtA, the three-dimensional structure of Ss-SrtA was simulated using Sp-SrtA as a template model whose structure has been deposited in the Protein Data Bank (PBD 3FN5). As shown in Fig 4B, the simulated Ss-SrtA structure was similar to that of Sp-SrtA. The overall structure adopted the same 8-stranded β-barrel fold unique to sortases [9, 10]. Sterically, C192 and R200 presented on the β7 and β8 strands, respectively, with H126 on a loop extending from the β4 strand. These three residues were sterically identical to the active site residues C208, R216 and H142 of Sp-SrtA [12], respectively. To functionally investigate the proposed roles of these three residues, the mutations H126A, C192A and R200A were constructed. All these mutations resulted in the loss of enzymatic activities against abz-LPATG-dnp, indicating that these three residues played critical roles in Ss-SrtA activity (data not shown). Taken together, H126, C192 and R200 were the key catalytic residues in Ss-SrtA.

### Residues K111 and D180 play key roles on Ca^2+^-independence of Ss-SrtA

Previous studies reveal that Ca^2+^ stimulates the activity of Sa-SrtA by 8-fold [20]. Ca^2+^ acts as an allosteric activator by stabilizing the structure of the β3/β4 loop and β6/β7 loop in Sa-SrtA in a closed, substrate-binding state. E105, E108, D112 and E171 are identified as the residues to which the Ca^2+^ binds [9–12, 23] (graphically shown in Fig 4C). In contrast to Sa-SrtA, the activities of Sp-SrtA and Ss-SrtA in this study were not dependent on Ca^2+^ [12]. Consistent with this finding, no structural equivalents in Sp-SrtA and Ss-SrtA were found for the four residues E105, E108, D112 and E171 in Sa-SrtA. A lysine (K126) was found to occupy the corresponding Ca^2+^ position in Sp-SrtA [23], but no further information for the Ca^2+^ independence of Sp-SrtA was provided. By structural alignment, we found that K111 on β3/β4 loop in Ss-SrtA was positioned at the same location as K126 in Sp-SrtA (Fig 4D). Further analysis revealed that D180 on β6/β7 loop was close enough to K111 to form a salt bridge. This salt bridge stabilized the closed state of the substrate contacting β6/β7 loop without assistance of extra Ca^2+^ ion, implying the mechanism of the Ca^2+^ independence of Ss-SrtA and Sp-SrtA. Sequence alignment revealed that these two residues were highly conserved in streptococcal sortases (Fig 4A, indicated by solid triangles). These findings suggested that all these streptococcal sortases might be Ca^2+^ independent.

### Acteoside, baicalin and isoquercitrin were newly identified as SrtA inhibitors

**Fig 5:**
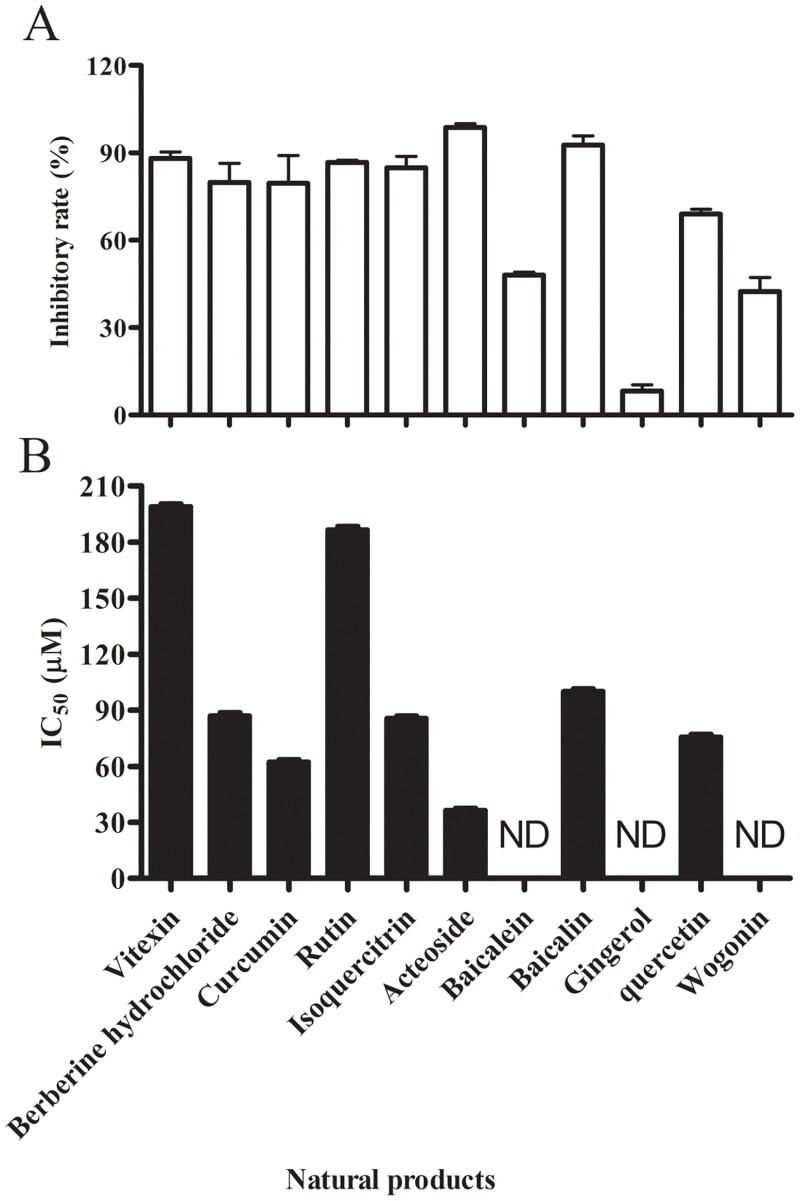
Inhibition of Ss-SrtA activity by natural products. A. Inhibitory rates of purified recombinant Ss-SrtA protein activity by natural products. B. IC_50_ detected for the natural products with inhibitory rates above 50%.

To find potent Ss-SrtA inhibitors, the 11 natural products listed in S1 Table were selected for experimental validation using purified recombinant Ss-SrtA. As shown in Fig 5A, 8 of these natural products strongly inhibited the activity of Ss-SrtA, showing >65% inhibitory rates at a concentration of 200 μM. Baicalein and wogonin slightly inhibited the activity of Ss-SrtA, showing 47% and 37% inhibitory rates, respectively, while gingerol barely affected the activity of Ss-SrtA. IC_50_ further indicated berberine hydrochloride, curcumin, isoquercitrin, acteoside, baicalin and quercetin were strong inhibitors for Ss-SrtA, with concentrations lower than 100 μM inhibiting >50% activity of Ss-SrtA (Fig 5B). Among these 6 natural compounds, berberine hydrochloride, curcumin, and quercetin have been determined to be strong SrtA inhibitors either with SrtA for *S*. *aureus* [24, 25] or *Streptococcus mutans* (*S*. *mutans*) [26], while isoquercitrin, acteoside, and baicalin are firstly identified as strong SrtA inhibitors in this study. Using the microtiter broth dilution method, we measured the MIC values of all these natural products to be >500 μM (S1 Table). These results indicate that these natural products do not function as antibiotics for *S*. *suis*.

### Acteoside strongly inhibited the activity of SrtA on *S*. *aureus* cells

**Fig 6:**
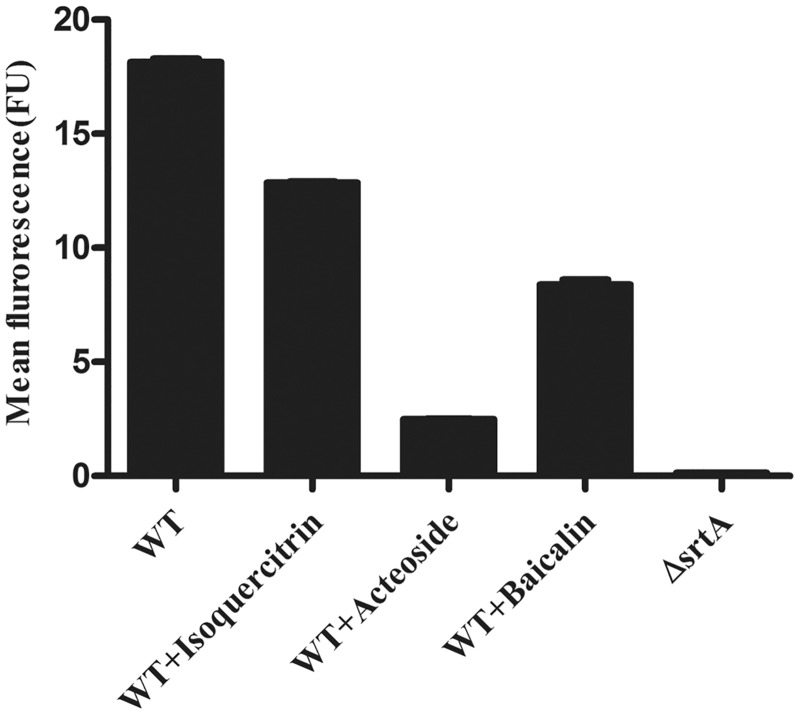
*In vivo* inhibition of staphylococcal sortase. After washing, late stationary grown *S*. *aureus* cells were incubated with 200 μM natural products for 1 h at 37°C and then incubated with 125 μM substrate 5 for 17 h. The mean fluorescence was determined by flow cytometry. Values for the *S*. *aureus* Δ*srtA* strain were included as a control.

Substrate 5 could be covalently incorporated into the cell wall of *S*. *aureus* in a SrtA-dependent manner [22]. However, we failed to observe the incorporation of substrate 5 into the cell wall of wildtype *S*. *suis* grown at different growth phases (data not shown). Because substrate 5 was originally designed on the basis of a natural substrate (a fibronectin-binding protein) for Sa-SrtA [22], we speculated that Ss-SrtA did not recognize substrate 5. Although sortases from different Gram-positive bacteria are structurally similar, the amino acid identity between sortases is limited to a few key residues at or near the active site [27]. Therefore, we asked whether these newly discovered inhibitors could inhibit Sa-SrtA in vivo. After 17h of incubation with substrate 5, wildtype *S*. *aureus* showed remarkable fluorescence that was associated with bacterial cells. This indicated that significant amounts of substrate 5 were incorporated into the cell wall. In contrast, limitd fluorescence was detected for *S*. *aureus* Δ*srtA* strain, which confirmed that the incorporation of substrate 5 was mainly mediated by endogenous SrtA (Fig 6). In agreement with the inhibitory effects of isoquercitrin, acteoside, and baicalin on Ss-SrtA protein, they all inhibited the incorporation of substrate 5 into the *S*. *aureus* cell wall by endogenous Sa-SrtA (Fig 6). However, the inhibitory rates of isoquercitrin and baicalin on endogenous Sa-SrtA (28% and 45%, respectively) were much lower than those on Ss-SrtA protein (81% and 95%, respectively). By contrast, acteoside exhibited high inhibitory rates on both endogenous Sa-SrtA (86%) and Ss-SrtA protein (98%), which suggested that this natural product was a potential inhibitor for SrtA from different Gram-positive bacteria.

## Discussion

*S*. *suis*, an emerging drug-resistant animal and human pathogen, may contribute to the spread of antibiotic resistance genes to streptococcal human pathogens such as *S*. *pyogenes*, *S*. *pneumoniae*, and *S*. *agalactiae*, acting as a resistance reservoir [3]. The identification of alternative therapeutic and preventive strategies against *S*. *suis* infections has become a priority. One strategy is to develop anti-infective therapies against gram-positive bacteria, focusing on small molecules that interfere with virulence protein regulation. SrtA has always been recognized as a potential target for anti-infective treatment and its inhibition represents a promising and effective strategy for the treatment and / or prevention of Gram-positive pathogen infections. In this study, Ss-SrtA was biochemically characterized and several natural products were screened as Ss-SrtA inhibitors.

Ss-SrtA, the product of open-reading frame SSU05_1074, exhibited approximately 60% identity with other reported streptococcal sortases. The purified recombinant Ss-SrtA had maximal activity at pH 6.0–7.5 and 45°C, stable at pH 6–9 and temperature lower than 45°C. The pH dependence was compatible with a reverse protonation mechanism [28]. These properties helped *S*. *suis* to anchor surface proteins to the cell wall in order to promote its survival in human or swine blood which had a pH close to 7.35–7.45 and a temperature of either 37°C or 41°C. Structural analysis and site-directed mutagenesis showed that H126, C192, and R200 were the key catalytic residues of Ss-SrtA, corresponding to residues H142, C208 and R216 in Sp-SrtA, respectively. The three residues were highly conserved in other Gram-positive bacteria. The *K*_m_ of Ss-SrtA to internally quenched fluorescent peptide substrate abz-LPATG-dnp was 6.7 μM, indicating it had higher affinity to abz-LPATG-dnp than Sa-SrtA, the *K*_m_ of which to the same substrate was 17.5 μM [29] These enzymatic characterizations provided useful information for future Ss-SrtA study.

Different from Sa-SrtA, whose activity was stimulated by Ca^2+^ [23], both Sp-SrtA and Ss-SrtA were found to be Ca^2+^ independent [12]. In Sa-SrtA, Ca^2+^ forms bonds with residues E105, E108, D112 and E171 to stabilize the structure of the β3/β4 loop and β6/β7 loop into a closed, substrate-binding state in Sa-SrtA [12]. Structural analysis revealed that a lysine (K111 in Ss-SrtA, K126 in Sp-SrtA) occupied the Ca^2+^ position observed in Sa-SrtA. We found that the D180 was near enough to K111 to form a salt bridge in Ss-SrtA. This aspartic acid residue was also conserved in Sp-SrtA. The salt bridge stabilized the structure of the β3/β4 loop and β6/β7 loop in Ss-SrtA and in Sp-SrtA, especially stabilized the structure at the closure of the substrate-binding cleft. This resulted a stable substrate-binding cleft in absence of Ca^2+^ in Ss-SrtA and Sp-SrtA. This explained why the activities of Ss-SrtA and Sp-SrtA were Ca^2+^ independent. Overall, the lysine at the position of K126 and the asparatic acid at the position of D180 in Ss-SrtA played key roles in the Ca^2+^ independence of Ss-SrtA. These two residues were highly conserved in other streptococcal sortases, suggesting all these sortases might be Ca^2+^ independent. The findings remain to be further confirmed by mutagenesis and ion effect studies.

Until recently, Indirect methods have been used to detect the presence of surface proteins in the cell wall to evaluate the inhibition of endogenous sortase activity by inhibitors, including protein A (SpA) and clumping factors A and B [29]. However, it did not account for the possibility that SrtA inhibitors might block the expression of these SrtA-anchored surface proteins. Very recently, a feasible approach was developed to incorporate a fluorescent-labeled synthetic peptide into the cell wall of *S*. *aureus* by endogenous SrtA [22]. As the amount of peptide incorporation was dependent on the activity of SrtA and could be conveniently detected by flow cytometry, it could also be a useful model for evaluating inhibitory effects on endogenous SrtA by inhibitors. In the present study, we utilized this model to determine the inhibitory effects of acteoside, baicalin, and isoquercitrin on Ss-SrtA activity on live cells. Unfortunately, *S*. *suis* did not incorporate this fluorescent peptide, substrate 5, into the cell wall as *S*. *aureus* did (data not shown) [22]. Because substrate 5 was designed based on a natural substrate (a fibronectin-binding protein) of Sa-SrtA, we speculated that substrate 5 might be not recognized by endogenous Ss-SrtA. Therefore, for future studies of Ss-SrtA, an appropriate fluorescent peptide substrate based on the natural substrates of Ss-SrtA, such as, SSads [30], SsnA [31] and Fbps [32], should be developed.

Over the past decade, natural products have been considered as a good source for screening SrtA inhibitors [13]. In this study, we screened Ss-SrtA inhibitors from 11 natural products, and found that berberine hydrochloride, curcumin, isoquercitrin, acteoside, baicalin and quercetin were strong inhibitors for purified recombinant Ss-SrtA. Berberine hydrochloride, curcumin, and quercetin have been determined to be strong SrtA inhibitors for *S*. *aureus* [24, 25] or *S*. *mutans* [26]. Our results revealed that these three natural products had lower inhibitory activities for *S*. *suis* sortase than that of *S*. *aureus* and *S*. *mutans*. Acteoside, baicalin and isoquercitrin were identified as strong SrtA inhibitors for the first time. When using this substrate 5-incorproation *S*. *aureus* model, unlike the strong inhibitory activities of isoquercitrin and baicalin on Ss-SrtA, much lower inhibitory rates were observed on endogenous Sa-SrtA (28% and 45%, respectively), indicating that these two inhibitors could not be widely used. By contrast, acteoside displayed high inhibitory rates on both endogenous Sa-SrtA (86%) and Ss-SrtA protein (98%), suggesting that such natural products were potential inhibitors for sortases from various Gram-positive pathogens.

By biochemical characterization, structural analysis and inhibitor screening, these results shed new light on enzymatic properties, Ca^2+^-independent catalytic mechanism and potential inhibitors of Ss-SrtA. These findings will contribute to the development of Ss-SrtA inhibitors, especially acteoside-based inhibitors.

## Supporting information

S1 Table Summary of 11 natural products screened in this study and their MIC values. (DOCX)
