# Supplementary material for: Streptococcus suis sortase A is Ca2+ independent and is inhibited by acteoside, isoquercitrin and baicalin
Source: PLoS One. 2017 Mar 20;12(3):e0173767. doi: 10.1371/journal.pone.0173767 (PMC5358767; doi:10.1371/journal.pone.0173767)
Supplement: S1 Table — (DOCX) [file pone.0173767.s001.docx]

**S1Table. Summary of 11 natural products screened in this study and their MIC values.**

|  | | | | |
| --- | --- | --- | --- | --- |
| **Compound** | **Structure** | **Molecular weight** | | **MIC** |
| Vitexin |  | 432 | >500μM | |
| Berberine hydrochloride |  | 371 | >500μM | |
| Curcumin |  | 368.38 | >500μM | |
| Rutin |  | 610.52 | >500μM | |
| Isoquercitrin |  | 464.38 | >500μM | |
| Acteoside |  | 624.59 | >500μM | |
| Baicalein |  | 270.24 | >500μM | |
| Baicalin |  | 446.36 | >500μM | |
| 6-Gingerol |  | 294.39 | >500μM | |
| quercetin |  | 302.24 | >500μM | |
| Wogonin |  | 284.07 | >500μM | |
